# Leveraging LASSO-based methodologies for enhanced SNP analysis in plant genomes

**DOI:** 10.1093/bioadv/vbaf014

**Published:** 2025-02-05

**Authors:** Nisha Puthiyedth, Farshad Zeinalinesaz, Dongdong Hou, Yue Zhang, Wenjun Lin, Yan Yan

**Affiliations:** Department of Computing Science, Thompson Rivers University, Kamloops, BC V2C 0C8, Canada; School of Computer Science, University of Guelph, Guelph, ON N1G 1Y4, Canada; School of Computer Science, University of Guelph, Guelph, ON N1G 1Y4, Canada; Department of Mathematics and Statistics, Thompson Rivers University, Kamloops, BC V2C 0C8, Canada; Digital Healthcare Innovation Lab, Faculty of Computer Science & Technology, Algoma University, Brampton, ON L6V 1A3, Canada; School of Computer Science, University of Guelph, Guelph, ON N1G 1Y4, Canada

## Abstract

**Summary:**

Genome-wide association studies (GWAS) have been widely used to reveal the associations between genetic variations and phenotypes in a population of individuals. However, they have been criticized for missing important genetic markers usually due to the fact that the data may not fit the statistical models well. In this study, we address the challenge of identifying significant single nucleotide polymorphisms (SNPs) in GWAS by harnessing the capabilities of two sophisticated regression models, BIGLASSO and AUTALASSO. They are both variants of the least absolute shrinkage and selection operator (LASSO). Our research contributes to the field of genomics through detailed comparative analysis of *Arabidopsis thaliana*, revealing how each method specializes in uncovering SNPs for different trait types. Our findings indicate that BIGLASSO shows stronger alignment with GWAS results, particularly excelling in the analysis of binary traits, even when these are derived from categorical phenotypes. AUTALASSO could be effective for quantitative traits and complement GWAS. We demonstrate that these LASSO-based methods can significantly enhance the identification of genetic markers, offering a potent complement to traditional GWAS approaches. Our findings not only bridge the gap between statistical and machine learning methodologies in genetic studies but also provide a practical framework for researchers seeking to validate reported SNPs or explore new genomic regions for trait association. This work stands as a pivotal step toward the integration of advanced computational techniques in genomics, paving the way for more precise and comprehensive genetic analyses.

**Availability and implementation:**

Key results from the paper are available at the https://github.com/DongdongHou006/LASSO-SNP. The program was implementated using Python and R, and was tested using the Digital Research Alliance of Canada.

## 1 Introduction

Genomics has experienced substantial development in recent years, especially with the advent of next-generation sequencing technologies. While these techniques offer a profusion of genetic data, interpreting meaningful patterns from them remains a challenge. Genome-wide association studies (GWAS) stand out as a powerful tool in this research field, enabling researchers to pinpoint genetic markers linked to complex traits. The commonly used genetic markers under study in GWAS are single nucleotide polymorphisms (SNPs), which are individual variations in DNA base pairs. GWAS perform statistical hypothesis tests for each SNP. From a GWAS experiment, SNPs along with their *P*-values are the typical output.

Many software packages have been developed to facilitate GWAS for genomic datasets, offering a range of methods for association analysis and genomic prediction/selection (GP/GS). Notable examples of these packages include PLINK (Purcell *et al.* 2007), TASSEL (Bradbury *et al.* 2007), GAPIT (Lipka *et al.* 2012), and GCTA (Yang *et al.* 2011), with PLINK being amongst the most revered.

The vast amount of SNPs from the whole-genome data brings significant challenges to GWAS models to correctly output SNPs associated with the traits with low *P*-values. In addition, different GWAS programs often produce dissimilar association results (Yan *et al.* 2019). One of the reasons for such challenges is the “large-*p*-small-*n*” in the data (Shen and Lin 2015), referring to datasets with more features than samples.

Advanced machine learning such as LASSO with its $l_{1}$ penalization (Tibshirani 2011), present promising avenues for addressing the challenges in “large-*p*-small-*n*” data. Some previous work of our team (Puliparambil *et al.* 2022a, 2022b) explored these challenges in the context of scRNA-seq data, and investigated the potential of using LASSO-based models to solve them.

LASSO is known for its feature selection capabilities which offer a valuable approach to enhance the analysis of genetic data. They promise to address overfitting, narrow down the feature set, and identify important SNPs influencing the phenotypes. Recently developed methods such as BIGLASSO (Zeng and Breheny 2020) and AUTALASSO (Waldmann *et al.* 2019) are examples of such applications in bioinformatics. They claim to have superior performance than the original LASSO. However, their prowess in deciphering plant genomes is yet to be exhaustively explored in existing literature.

This research tries to investigate LASSO-based models on plant genome analysis to find out significant SNPs related to the trait of interest. Our objective is to enrich the genetic research toolkit by evaluating LASSO-derived methodologies in a model plant, *Arabidopsis thaliana*, and contrasting their outcomes with the well-established GWAS software, PLINK. Additionally, we have included other GWAS programs, such as TASSEL (Bradbury *et al.* 2007), GAPIT (Tang *et al.* 2016), and GCTA (Yang *et al.* 2011), to compare their outcomes with those generated by AUTALASSO and BIGLASSO for different types of phenotypes including binary, categorical, and quantitative traits. Our contributions bridge contemporary techniques with traditional GWAS, potentially reshaping the modalities of extracting insights from plant genomes.

## 2 Methods

**Figure 1.**
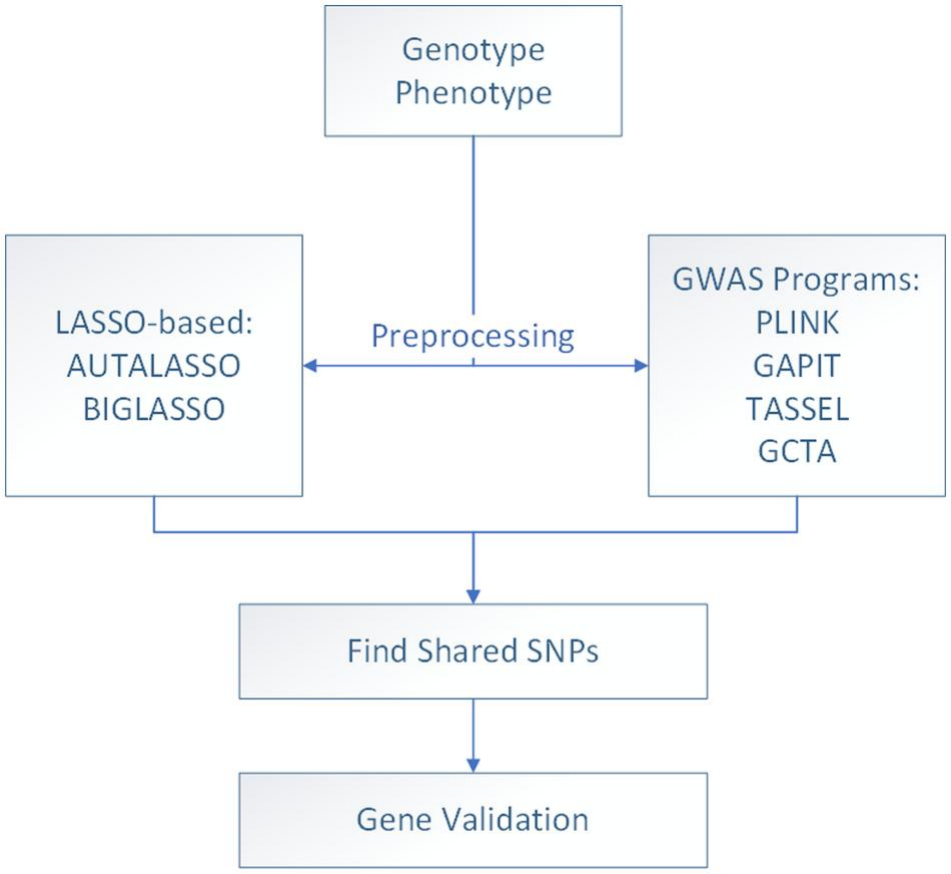
Visual representation of the study’s workflow.

This research undertakes a systematic approach to understand and compare the capabilities of BIGLASSO and AUTALASSO in SNP identification using genotypic and phenotypic data. The output from BIGLASSO and AUTALASSO are further compared with four GWAS programs including PLINK, TASSEL, GAPIT, and GCTA. The workflow from data selection to SNP identification and gene association is illustrated in Fig. 1. Detailed descriptions of all models and analysis methods are as follows.

### 2.1 BIGLASSO

BIGLASSO, a specialized R package, is tailored for managing substantial datasets (such as whole-genome SNP data), often exceeding available RAM capacity. It is the implemented version of LASSO and Elastic Net penalties with sparse linear and logistic regression models. The coefficient estimates for big lasso can be obtained as follows:


(1) $${\hat{\beta }}_{BL}=\arg\underset{\beta }{\min}\left(\frac{1}{n}{\left\|\gamma -X\beta \right\|}^{2}+\lambda {\left\|\beta \right\|}_{1}\right)$$

where the tuning parameter is λ≥0, which controls the amount of regularization to be injected.

BIGLASSO’s features include:

- Efficient memory-mapping techniques.
- Utilization of swift pathwise coordinate descent and “warm start” strategies.
- Incorporates hybrid feature screening rules.
- Conserves memory by employing “cell-wise standardization”.
- Support for parallel computing, boosting computational speeds.

Considering the benefits of BIGLASSO, we have chosen to utilize it in our research.

### 2.2 AUTALASSO

AUTALASSO incorporates the alternating direction method of multipliers (ADMM) optimization algorithm, as proposed by Boyd *et al.* (2010). This integration ensures automatic hyper-parameter tuning, encompassing learning rates and regularization factors, which is particularly advantageous for adaptive LASSO (Huang *et al.* 2008). The method maintains a delicate balance between accuracy and computational efficiency. In the original study by Waldmann *et al.* (2019), which introduced AUTALASSO, the model underwent rigorous testing on both simulated and real datasets. The framework demonstrated its capability for swift convergence and efficient model training, establishing its suitability for large-scale datasets commonly encountered in genomics research.

Nonetheless, it is worth noting that the application of AUTALASSO to plant genome data remains unexplored. Thus, our objective is to bridge this knowledge gap by employing AUTALASSO to assess binary, quantitative, and categorical phenotypes in plant genome data.

### 2.3 GWAS programs

Here, we give a detailed description on all GWAS programs used in this study.

Originally designed for the human genome, PLINK’s adaptability has cemented its status in diverse genetic studies. It was one of the earliest packages for GWAS and is viewed as a standard method. PLINK offers users two interface options: a command-line program and a Java-based graphical user interface (GUI) called gPLINK, which provides access to the most frequently used PLINK commands. This versatile tool can process both text files (accessed using the –file flag) and binary files (accessed using the –bfile flag). PLINK’s capabilities extend to various essential tasks in genetic analysis, including preliminary data quality control (QC) and association testing.

TASSEL is an open-source, cross-platform software developed in Java, specifically designed for genetic analysis with a focus on maize. It supports two primary statistical models for association studies: the general linear model (GLM) and the mixed linear model (MLM). In addition to these core capabilities, TASSEL offers a range of supplementary functions. These include assessing genetic relationships between samples, measuring linkage disequilibrium, performing principal component analysis to understand population structure, clustering samples based on genetic similarity, imputing missing genotype data, and visualizing genetic data. With its comprehensive set of tools tailored for maize genetics and genomics, TASSEL serves as a powerful and flexible platform for plant geneticists to analyse their data.

GAPIT is a versatile tool that provides a wide array of methods for GWAS and genomic selection (GS) within the R software environment. It supports various input data formats, including numeric, hapmap, and PLINK genotype formats, and generates comprehensive reports that are publication-ready. When conducting GWAS with multiple traits, environments, or models, GAPIT produces integrated Manhattan plots with highlighted associated markers for easy interpretation. The required input data consists of phenotype (Y), genotype in hapmap format (G), numerical genotype data (GD), genotype map (GM), kinship (K), and covariate variables (CV), with phenotypic data being mandatory. The software supports various modules including MLM, GLM, CMLM, MLMM, FarmCPU, and BLINK. These models offer different levels of statistical power, with FarmCPU outperforming MLMM and BLINK surpassing FarmCPU in GWAS analysis. In single locus models, MLM is superior to GLM, and CMLM outshines MLM. The focus of this article is on MLM results, while the results of other methods like BLINK are considered future work.

GCTA stands for genome-wide complex trait analysis. It addresses the “missing heritability” problem by estimating variance explained by all SNPs for complex traits. It offers MLM analysis as a key feature. GCTA provides two main MLM-based association analysis options. The –mlma option performs standard MLM analysis including the candidate SNP. The –mlma-loco option implements a “leave-one-chromosome-out” analysis, excluding the chromosome with the candidate SNP from GRM calculation. This LOCO approach can provide increased statistical power compared to the standard MLM, albeit at a higher computational cost. These features make GCTA a versatile tool for analysing complex genetic traits in large GWAS datasets. In this study, we will present findings from both the –mlma and –mlma-loco analyses.

### 2.4 Comparative SNP analysis

The output of BIGLASSO and AUTALASSO consists of lists of SNPs. The results from lasso-based methods come with coefficient values that can be used as the significance. In contrast, traditional GWAS outputs SNPs with associated *P*-values signifying their significance. To facilitate a meaningful comparison, the outputs from both lasso-based models were cross-referenced against GWAS results. For this study, we utilized a suite of widely recognized tools in GWAS methodology, including PLINK, TASSEL, GAPIT, and GCTA. These tools are considered standards in the field and were selected to ensure robust and comprehensive analysis. This comparison aims to provide a comprehensive understanding of the relative advantages and limitations of the two lasso-based approaches in comparison to a well-established GWAS methods.

### 2.5 Experimental data and settings

#### 2.5.1 Experimental dataset

To perform GWAS and compare their output SNPs with the output from the above two LASSO-based methods, an independent dataset is used. Since we focus on plant genomes in this study, a model plant *A. thaliana* dataset named AtPolyDB is chosen.

The AtPolyDB dataset is available from the easyGWAS website and originated from two papers of GWAS on *A. thaliana* (Atwell *et al.* 2010, Horton *et al.* 2012). It has 1307 samples with 214 051 SNPs and 107 different phenotypes, though not every sample has 107 phenotypes. The phenotypes include binary, quantitative, and categorical ones. Two binary (Emco5 and Anthocyanin_22) and two quantitative phenotype [flowering time at 10°C (FT10) and Width_22] are selected because they both have a large number of samples. Three categorical phenotypes [silique length at 16°C (Silique_16), silique length at 22°C (Silique_22), and days to germination at 22°C (Germ_22)] are also selected. Note that not all phenotypes contain all 1307 attributes and some contain null values; these null values were removed during the pre-processing phase. There were no missing SNPs in the genotype data. Detailed information about the experimental data is shown in Table 1.

**Table 1.** Overview of experimental data from AtPolyDB dataset.

Phenotype	Type	#Samples	Description
Emco5	Binary	86	Protist disease resistance
Anthocyanin_22	Binary	177	Visual anthocyanin presence
FT10	Continuous	194	Flowering time at 10°C
Width_22	Continuous	175	Plant diameter
Silique_16	Categorical	95	Silique length
Silique_22	Categorical	95	Silique length
Germ_22	Categorical	177	Germination days at 22°C

#### 2.5.2 Pre-processing and parameter settings

The pre-processing step is essential to ensure data compatibility with LASSO-derived methodologies. First, we convert the chromosomal nucleotide information into numeric values. Next, we incorporate the phenotypes into the genotypes to create an input file in a format suitable for Lasso-based methods. Additionally, we remove samples with missing phenotype values.

For BIGLASSO, our preliminary study showed that the method reported errors for the quantitative phenotypes. After some further investigation into this issue, we realized that it may only handle binary phenotypes in the model. Therefore, we transform the phenotypic data of the continuous phenotypes (FT10 and Width_22) and categorical phenotypes (Silique_16, Silique_22, and Germ_22) into binary values for analysis. In BIGLASSO method, the choice of the family argument depends on the response vector. Since we used the binary variations, the family argument is used as a binomial in the “biglasso” R package (Zeng and Breheny 2020) for both phenotypes. Other settings are kept as default.

To compare the results from BIGLASSO and AUTALASSO with GWAS, we set the threshold of the number of SNPs as 20. The number of Biglasso results depends on the Lambda parameter, so the Lambda value closest to output 20 SNPs is chosen for model training. In this way, a reasonable number of top-ranked SNPs can be compared without exploring the total list of SNPs (in our case 214 051). It is often a common practice in genomic studies to further investigate only the SNPs that passed a certain *P*-value threshold from GWAS, or select some top-ranked ones. Another reason for setting up this threshold is that BIGLASSO outputs 20 SNPs as important ones. For AUTALASSO, we use the absolute coefficient values to select the top 20 SNPs, and for GWAS, we use *P*-values to rank SNPs.

## 3 Results

In this section, we demonstrate the performance of BIGLASSO and AUTALASSO on the AtPolyDB dataset with the selected phenotypes, and compare them with the GWAS programs PLINK, TASSEL, GAPIT, and GCTA.

### 3.1 GWAS and LASSO-based methods on Emco5 phenotype

BIGLASSO was applied to the AtPolyDB dataset, and the 20 significant SNPs were output based on the regularization criterion within the “biglasso” R package. These SNPs were then used for further comparison with PLINK, TASSEL, GAPIT, and GCTA.

Results showed that there was a high agreement between the SNPs from BIGLASSO, PLINK, and GAPIT. Part of the results is captured in Table 2. We can see from Table 2 that the top three SNPs identified by BIGLASSO (Chr2_2990590, Chr1_1430179, and Chr5_20112353) are also ranked among the top three by PLINK. Additionally, the top two SNPs from BIGLASSO (Chr2_2990590 and Chr1_1430179) match exactly with the top two SNPs identified by GAPIT. In addition, eight of the 20 reported SNPs by BIGLASSO are also within the top 20 ranked SNPs by PLINK and GAPIT. All those SNPs have very small *P*-values from PLINK indicating they are associated with the trait with high confidence.

**Table 2.** Comparison of BIGLASSO, PLINK, and GAPIT results for AtPolyDB dataset, phenotype Emco5.

Chromosome	SNP ID	PLINK rank	GAPIT rank
Chr2	Chr2_2990590	1	1
Chr1	Chr1_1430179	3	2
Chr5	Chr5_20112353	2	9
Chr5	Chr5_21697730	6	6
Chr5	Chr5_21041671	17	5
Chr5	Chr5_22625167	8	
Chr4	Chr4_783577	16	8
Chr3	Chr3_9028198		10
Chr3	Chr3_14993958	18	
Chr1	Chr1_4266002	10	14
Chr5	Chr5_2232651	5	7
Chr5	Chr5_13945171		4
Chr2	Chr2_155225	11	

Shows chromosome number, SNP ID with position, and corresponding ranks in PLINK and GAPIT outputs. Empty cells indicate no ranking found for that SNP in the respective tool.

The results from BIGLASSO, PLINK, and GAPIT on Emco5 phenotype demonstrate a strong consensus among all three models on the top 20 SNPs, and they could be used as a verification increasing the confidence that those eight SNPs are potentially contributing to the phenotype. Meanwhile, no shared SNPs were observed between BIGLASSO and TASSEL or GCTA, possibly due to the distinct modeling methods used. BIGLASSO employs penalized models, whereas TASSEL and GCTA utilize the MLM.

**Figure 2.**
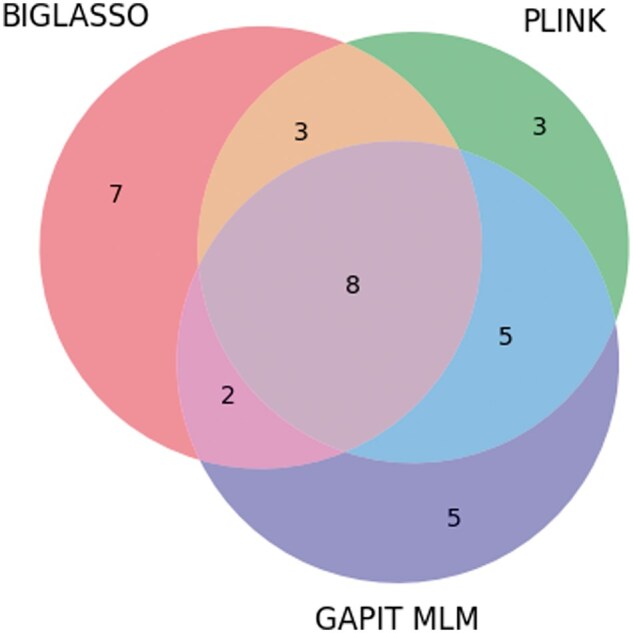
Venn diagram showing the number of overlap SNPs between BIGLASSO, PLINK, and GAPIT on Emco5 phenotype.

The number of overlapping SNPs among BIGLASSO, PLINK, and GAPIT on Emco5 phenotype is shown in Fig. 2.

When AUTALASSO was applied to the Emco5 phenotype, it failed to output any SNPs. After further investigation, we found that it may have limitations on binary phenotypes. In our study, AUTALASSO is then only used for the quantitative and categorical phenotype.

### 3.2 GWAS and LASSO-based methods on Anthocyanin_22 phenotype

When BIGLASSO was applied to another binary phenotype: Anthocyanin_22, 19 significant SNPs were output based on the regularization criterion within the “biglasso” R package. These SNPs were then used for further comparison with the GWAS software (PLINK, TASSEL, GAPIT, and GCTA).

**Figure 3.**
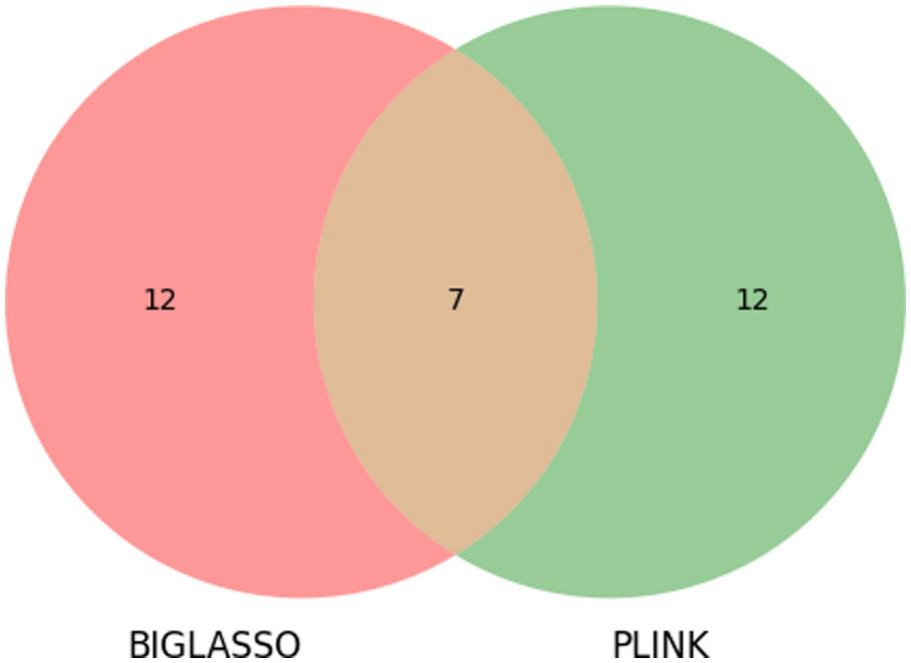
Venn diagram showing the number of overlap SNPs between BIGLASSO and PLINK on Anthocyanin_22 phenotype.

**Figure 4.**
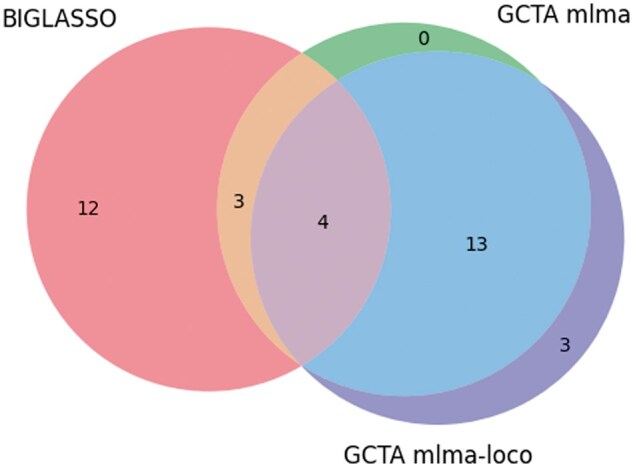
Venn diagram showing the number of overlap SNPs between BIGLASSO and GCTA models (mlma and mlma-loco) on Anthocyanin_22 phenotype.

The result in Table 3 demonstrated a strong concordance between the SNPs identified by BIGLASSO, PLINK, and GCTA for the Anthocyanin_22 phenotype. We can observe from Table 3 that the top two ranked SNPs identified by BIGLASSO (Chr1_14007403, Chr1_16930622) are consistent with the top two ranked SNPs reported by PLINK. Additionally, 10 of the 19 reported SNPs by BIGLASSO are also within the top 20 ranked SNPs by GWAS software. Furthermore, BIGLASSO, PLINK, and GCTA all identified two common SNPs (Chr4_13110999 and Chr4_1503923). This overlap underscores the consistency of the results from BIGLASSO across these different methods. The numbers of overlapping SNPs between BIGLASSO and PLINK, and between BIGLASSO and GCTA are also shown in Figs 3 and 4.

**Table 3.** Comparison of BIGLASSO with PLINK and GCTA for AtPolyDB dataset, phenotype Anthocyanin_22.

Chromosome	SNP ID	PLINK rank	GCTA rank	GCTA LOCO rank
Chr1	Chr1_14007403	2		
Chr1	Chr1_16930622	1		
Chr4	Chr4_13110999	12	6	2
Chr1	Chr1_4550338	16		
Chr4	Chr4_1503923	3	13	9
Chr4	Chr4_7976992	14	2	
Chr1	Chr1_5058178		4	8
Chr2	Chr2_18985764		1	
Chr1	Chr1_8722354		17	13
Chr4	Chr4_7981082	15	3	

Columns show chromosome number, SNP ID with position, and rankings from PLINK, GCTA(mlma), and GCTA(mlma-loco). Empty cells indicate no ranking found for that SNP in the respective tool.

The results from BIGLASSO, PLINK, and GCTA on Anthocyanin_22 phenotype show that these models have high agreement on the top 20 SNPs, and they could be used as a verification increasing the confidence that those overlapping SNPs are potentially contributing to the phenotype.

When AUTALASSO was applied to the Anthocyanin_22 phenotype, which is a binary phenotype, it failed to output any SNPs.

### 3.3 GWAS and LASSO-based methods on FT10 and Width_22 phenotypes

When applying BIGLASSO to the FT10 and Width_22 phenotypes, we converted them to binary values so that they can be processed by BIGLASSO. We found that BIGLASSO and GWAS results shared some SNPs on the FT10 and Width_22 phenotypes, as shown in Tables 4 and 5.

**Table 4.** Comparison of BIGLASSO with PLINK and GCTA for AtPolyDB dataset, phenotype FT10.

SNP ID	PLINK rank	GAPIT rank	GCTA rank	GCTA LOCO rank
Chr2_14324385	1			
Chr2_7434280	3			
Chr5_21579246	15			
Chr5_26691411	5			
Chr1_18860998	13			
Chr5_23252765				
Chr4_12101415	10			
Chr4_15930433	6			
Chr2_9604507	2		19	
Chr5_18600065	17			
Chr1_16188446				
Chr3_5439732				
Chr4_12210810	12			
Chr2_1921175				
Chr4_529409	18	8		5
Chr4_424108		15		
Chr4_429928				
Chr2_14209647				
Chr4_424505				
Chr1_566941				
Chr4_454542			20	11

Columns show SNP ID with position, and rankings from PLINK, GAPIT, GCTA(mlma), and GCTA(mlma-loco). Empty cells indicate no ranking found for that SNP in the respective tool.

**Table 5.** Comparison of BIGLASSO with PLINK and GCTA for AtPolyDB dataset, phenotype Width_22.

Chromosome	SNP ID	PLINK rank
Chr3	Chr3_11654990	1
Chr5	Chr5_18098467	14
Chr3	Chr3_8200474	
Chr3	Chr3_7646303	
Chr3	Chr3_14593730	17
Chr5	Chr5_16825143	
Chr3	Chr3_15742125	3
Chr4	Chr4_17569323	
Chr4	Chr4_9274831	9
Chr4	Chr4_8066659	2
Chr1	Chr1_12349397	18
Chr5	Chr5_22416214	
Chr1	Chr1_7373779	4
Chr4	Chr4_3831266	
Chr1	Chr1_23976183	
Chr3	Chr3_16227974	
Chr1	Chr1_27611078	
Chr5	Chr5_9176769	6
Chr4	Chr4_16460215	7
Chr5	Chr5_15873167	
Chr5	Chr5_18098575	15
Chr5	Chr5_18099838	16

Columns show Chromosome, SNP ID with position, and rankings from PLINK, GAPIT, GCTA(mlma), and GCTA(mlma-loco). Empty cells indicate no ranking found for that SNP in the respective tool.

AUTALASSO, when applied to the AtPolyDB dataset with the FT10 and Width_22 phenotypes, selected the top 18 SNPs based on the absolute coefficient values. A comparison with PLINK results showed a lack of agreement, with the SNPs from AUTALASSO having large *P*-values from PLINK and ranked outside of the top 20. We further conducted comparisons between the results obtained from AUTALASSO and other GWAS programs such as TASSEL, GAPIT, and GCTA, revealing that they lacked agreement with those programs as well. One side note on the GWAS analysis on the same data showed that they did not have a high agreement as well [detailed results in our previous study comparing GWAS programs (Yan *et al.* 2019)]. This could indicate that these particular phenotypes are sensitive to models, or they are complicated and no one method could catch all potential SNPs, or some other reasons. The concrete conclusion may need further investigation and it is worthwhile for future studies.

Given these differences between GWAS and LASSO-based methods on FT10 and Width_22 phenotypes, we examined deeper into the SNPs output from BIGLASSO and AUTALASSO. We would like to examine those SNPs’ potential linkage to genes affecting the FT10 phenotype (Flowering time at 10°C with 16 h daylight) and Width_22 (plant width). Using The Arabidopsis Information Resource (TAIR), we searched for genes within a ±1000 bp radius of each reported SNP. Our analysis revealed that BIGLASSO identified SNPs associated with four distinct genes related to growth and development for the FT10 phenotype. Interestingly, AUTALASSO linked nine genes to the FT10 phenotype, all of which were expressed either during the flowering stage or in flowers. These findings are summarized in Tables 6 and 7. We conducted the same analysis for the Width_22 phenotype using both BIGLASSO and AUTALASSO. The results, included in Tables 8 and 9, show that BIGLASSO identified 14 genes linked to Width_22, while AUTALASSO identified six genes associated with this phenotype.

**Table 6.** Genes and their annotations linked to the SNPs reported by BIGLASSO on FT10 phenotype.

SNP	Genes	Annotation and functions
Chr2_14324385	ETT	Floral meristem determinacy
Chr5_23252765	AT5G57390	Essential for the developmental transition between the embryonic and vegetative phases in plants
Chr4_429928	AT4G00990	Loss of function mutants are susceptible to bacterial infection and early flowering
Chr2_14209647	AT2G33540	Acts during flowering to dephosphorylate FLX4 which in turn promotes FLC expression

These genes have functions closely related to flower, leaf, or tissue growth. SNP is the ID of the SNP from the dataset. The number after the dash is the position of the SNP. Genes denote the corresponding genes to the SNPs selected. Annotation and functions represent the functions of the associated genes.

**Table 7.** Genes and their annotations linked to the SNPs reported by AUTALASSO on FT10 phenotype.

SNP	Genes	Annotation and functions
Chr1_8435789	AT1G23870	Encodes an enzyme putatively involved in trehalose biosynthesis
Chr1_8435789	AT1G23880	NHL domain-containing protein
Chr1_24285106	AT1G65370	TRAF-like family protein
Chr1_26152060	AT1G69550	Disease resistance protein
Chr5_2166149	AT5G06970	PATROL1 is a Munc13-like protein involved in mediating H[+]-ATPase translocation
Chr5_3451333	AT5G10940	ASG2 is farnesylated protein and this post-translational modification impacts its subcellular localization
Chr5_14561676	AT5G36910	Encodes a thionin that is expressed at a low basal level in seedlings and shows circadian variation
Chr5_15489291	AT5G38700	Cotton fiber protein
Chr5_22985902	AT5G56850	Hypothetical protein

These genes have functions closely related to flower, leaf, or tissue growth. SNP is the ID of the SNP from the dataset. The number after the dash is the position of the SNP. Genes denote the corresponding genes to the SNPs selected. Annotation and functions represent the functions of the associated genes.

**Table 8.** Genes and their annotations linked to the SNPs reported by BIGLASSO on Width_22 phenotype.

SNP	Genes	Annotation and functions
Chr5_18098467	AT5G44820	Nucleotide-diphospho-sugar transferase family protein
Chr3_8200474	AT3G23060	RING/U-box superfamily protein
Chr3_7646303	AT3G21700	Expressed during pollen development and in the pollen tube tip
Chr5_16825143	AT5G42080	Result in defects in embryogenesis, cell plate formation and trichome branching
Chr4_9274831	AT4G16444	ER localized protein that Interacts with GET3a and GET2 orthologs
Chr1_12349397	AT1G33960	Identified as a gene that is induced by avirulence gene avrRpt2 and RPS2
Chr1_12349397	AT1G33970	Its expression is repressed upon pathogen infection
Chr5_22416214	AT5G55260	Encodes a protein with similarity to the catalytic subunit of the mammalian PPX protein phospatase
Chr1_7373779	AT1G21060	Serine/threonine-kinase, putative
Chr1_7373779	AT1G21065	Secondary thiamine-phosphate synthase enzyme
Chr3_16227974	AT3G44680	Functions in promoting the onset of leaf senescence.
Chr1_27611078	AT1G73430	Involved in tethering of retrograde intra Golgi vesicles
Chr1_27611078	AT1G73440	Calmodulin-like protein
Chr5_18098575	AT5G44820	Nucleotide-diphospho-sugar transferase family protein

SNP is the ID of the SNP from the dataset. The number after the dash is the position of the SNP. Genes denote the corresponding genes to the SNPs selected. Annotation and functions represent the functions of the associated genes.

**Table 9.** Genes and their annotations linked to the SNPs reported by AUTALASSO on Width_22 phenotype.

SNP	Genes	Annotation and functions
Chr1_22863232	AT1G61860	Protein kinase superfamily protein
Chr1_22863232	AT1G61850	Mutant plants were impacted in resistance to fungus B
Chr2_697735	AT2G02560	Ubiquitously expressed in plant tissues throughout development
Chr2_697735	AT2G02570	Similar to SPF30 splicing factor
Chr2_697947	AT2G02560	Displayed developmental phenotypes similar to downwardly curling leaves
Chr5_17716713	AT5G44030	Encodes a cellulose synthase involved in secondary cell wall biosynthesis

SNP is the ID of the SNP from the dataset. The number after the dash is the position of the SNP. Genes denote the corresponding genes to the SNPs selected. Annotation and functions represent the functions of the associated genes.

Results from Tables 6 to 9 indicate that LASSO-based methods could identify regions containing genes relevant to the phenotype of interest. The disagreement with GWAS suggests that some of these regions might be overlooked in traditional GWAS analysis. Therefore, LASSO-based methods could potentially complement GWAS in this case, offering new genomic regions and genes for further examination.

### 3.4 GWAS and LASSO-based methods on Silique_16, Silique_22, and Germ_22 phenotypes

We applied BIGLASSO to the categorical phenotypes Silique_16, Silique_22, and Germ_22 by transforming these traits into binary values based on their respective mean values (to satisfy BIGLASSO’s requirements). Results on shared SNPs between BIGLASSO and GWAS are demonstrated in Tables 10–12 for Silique_16, Silique_22, and Germ_22 phenotypes, respectively.

**Table 10.** Comparison of BIGLASSO with PLINK and GCTA for AtPolyDB dataset, phenotype Silique_16.

Chromosome	SNP ID	PLINK rank
Chr1	Chr1_25433664	
Chr2	Chr2_1259706	8
Chr1	Chr1_5869785	
Chr3	Chr3_8298944	
Chr3	Chr3_20381442	
Chr2	Chr2_7393715	1
Chr3	Chr3_23315915	4
Chr5	Chr5_230495	6
Chr1	Chr1_18339386	7
Chr2	Chr2_6813344	3
Chr1	Chr1_7812463	
Chr4	Chr4_6354389	9
Chr5	Chr5_25915288	5
Chr5	Chr5_4672003	
Chr1	Chr1_4022922	
Chr3	Chr3_16126394	20
Chr1	Chr1_6909432	
Chr5	Chr5_22192979	
Chr1	Chr1_8431840	
Chr4	Chr4_9767968	
Chr5	Chr5_17378017	
Chr2	Chr2_7393754	2

Columns show Chromosome, SNP ID with position, and rankings from PLINK. Empty cells indicate no ranking found for that SNP in the respective tool.

**Table 11.** Comparison of BIGLASSO with GCTA for AtPolyDB dataset, phenotype Silique_22.

Chromosome	SNP ID	GCTA rank	GCTA_LOCO rank
Chr1	Chr1_7933107		
Chr1	Chr1_16834866		
Chr5	Chr5_17242464		
Chr5	Chr5_10025947		
Chr3	Chr3_4455708		
Chr4	Chr4_5717021		
Chr4	Chr4_10796353		
Chr5	Chr5_15098822		
Chr1	Chr1_16832623		
Chr3	Chr3_14747160		
Chr5	Chr5_20096064		
Chr3	Chr3_11522549		
Chr2	Chr2_3942724	16	2
Chr1	Chr1_9777123		
Chr5	Chr5_22136038		
Chr5	Chr5_18001211		
Chr3	Chr3_2775964		
Chr4	Chr4_8298259		
Chr3	Chr3_15093392		
Chr4	Chr4_5717864		

Columns show chromosome number, SNP ID with position, and rankings from GCTA(mlma), and GCTA(mlma-loco). Empty cells indicate no ranking found for that SNP in the respective tool.

**Table 12.** Comparison of BIGLASSO with GCTA for AtPolyDB dataset, phenotype Germ_22.

Chromosome	SNP ID	GCTA rank	GCTA_LOCO rank
Chr2	Chr2_3853806		
Chr3	Chr3_16905844		
Chr1	Chr1_17913720		
Chr4	Chr4_10411003		
Chr5	Chr5_7162596	12	11
Chr2	Chr2_19474849		
Chr3	Chr3_14789506		
Chr5	Chr5_17165413		
Chr4	Chr4_12294772		
Chr4	Chr4_17180542		
Chr4	Chr4_17025614		
Chr1	Chr1_16740979		
Chr5	Chr5_15766333		
Chr3	Chr3_2564078		
Chr1	Chr1_6061258		
Chr2	Chr2_19439970		
Chr3	Chr3_12652072		
Chr2	Chr2_19416941		
Chr3	Chr3_14912580		
Chr5	Chr5_5912851	19	14
Chr3	Chr3_14783947		

Columns show chromosome number, SNP ID with position, and rankings from GCTA(mlma), and GCTA(mlma-loco). Empty cells indicate no ranking found for that SNP in the respective tool.

**Figure 5.**
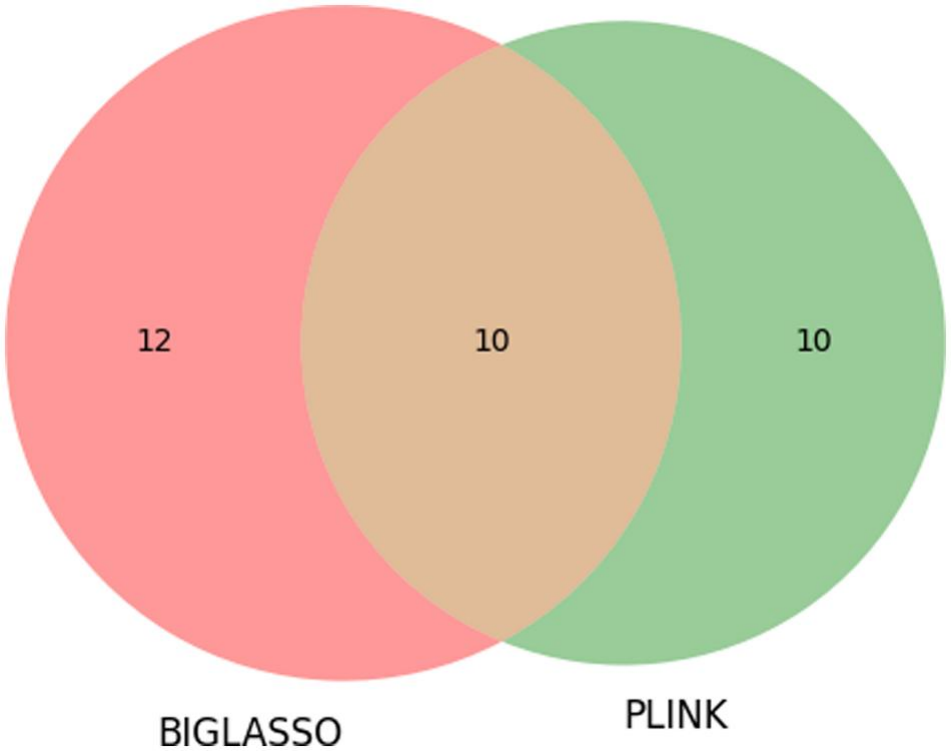
Venn diagram showing the number of overlap SNPs between BIGLASSO and PLINK on Silique_16 phenotype.

We can observe from Table 10 that 10 out of the 22 SNPs identified by BIGLASSO are also among the top 20 ranked SNPs reported by PLINK. The alignment between the BIGLASSO and PLINK findings provides a form of cross-validation, reinforcing the credibility of the overlapping SNPs associated with the Silique_16 phenotype. The intersection of these findings is visually represented in Fig. 5. Additionally, the SNPs identified by BIGLASSO do not overlap with results from other GWAS software we investigated including GAPIT, TASSEL, and GCTA.

**Figure 6.**
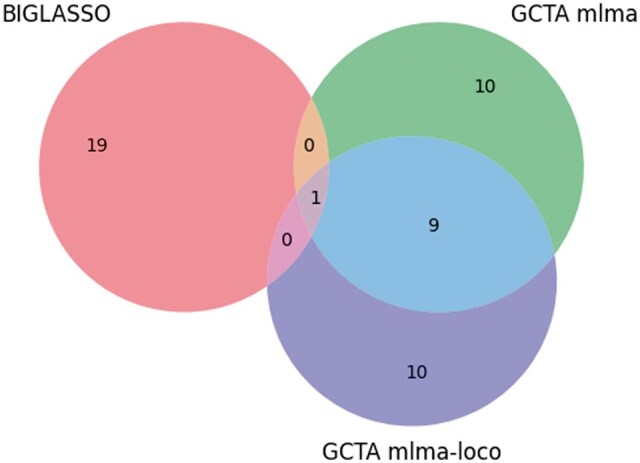
Venn diagram showing the number of overlap SNPs between BIGLASSO and GCTA on Silique_22 phenotype.

**Figure 7.**
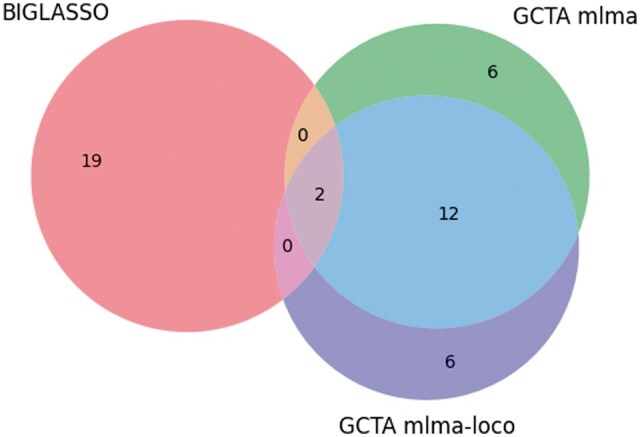
Venn diagram showing the number of overlap SNPs between BIGLASSO and GCTA on Germ_22 phenotype.

Notably, as shown in Tables 11 and 12, the SNPs identified by BIGLASSO for the Silique_22 and Germ_22 phenotypes demonstrate very little overlap with the top 20 SNPs identified by GWAS methods, sharing only one or two SNPs in common with the results from GCTA. This disparity is visually represented in Figs 6 and 7, which illustrate the limited intersection between BIGLASSO and GWAS results for Silique_22 and Germ_22 phenotypes, respectively. The contrasting results for Silique_22 and Germ_22 compared to Silique_16 suggest that BIGLASSO’s performance may vary depending on the specific phenotype. Future research is need to explore specific conclusions. Additionally, alternative methods for converting categorical phenotypes to binary formats should be investigated, as these may affect the results obtained from BIGLASSO.

To further elucidate the biological significance of the SNPs identified by BIGLASSO, we conducted a comprehensive investigation of their genomic context. Leveraging TAIR, we analysed the genes located within a ±1000 bp radius of each reported SNP to demonstrate their association with relevant phenotypes. This analysis revealed that BIGLASSO identified SNPs associated with several distinct genes related to growth and development for the Silique_22 (Silique length) and Germ_22 (germination days at 22°C) phenotypes. Detailed information regarding these associations is presented in Tables 13–15. The discovery of SNPs by BIGLASSO that were not detected by GWAS methods highlights its potential in SNPs identification.

**Table 13.** Genes and their annotations linked to the SNPs reported by BIGLASSO on Silique_16 phenotype.

SNP	Genes	Annotation and functions
Chr1_25433664	AT1G67830	Expressed during plant embryo bilateral stage
Chr1_25433664	AT1G67840	Expressed during plant embryo cotyledonary stage
Chr1_5869785	AT1G17160	Expressed during mature plant embryo stage
Chr1_5869785	AT1G17170	Expressed in carpel
Chr1_8431840	AT1G23870	Expressed during mature plant embryo stage and expressed in carpel
Chr2_1259706	AT2G03980	Expressed during plant embryo cotyledonary stage
Chr2_6813344	AT2G15620	Expressed in carpel plant embryo
Chr3_20381442	AT3G55000	Expressed during mature plant embryo stage
Chr3_23315915	AT3G63090	Expressed in carpel
Chr3_16126394	AT3G44540	Expressed in carpel, plant embryo
Chr4_9767968	AT4G17510	Expressed during plant embryo cotyledonary stage
Chr5_230495	AT5G01600	Expressed during mature plant embryo stage
Chr5_230495	AT5G01610	Expressed during mature plant embryo stage
Chr5_25915288	AT5G64830	Expressed in carpel, plant embryo
Chr5_22192979	AT5G54630	Expressed during plant embryo cotyledonary and globular stage

SNP is the ID of the SNP from the dataset. The number after the dash is the position of the SNP. Genes denote the corresponding genes to the SNPs selected. Annotation and functions represent the functions of the associated genes.

**Table 14.** Genes and their annotations linked to the SNPs reported by BIGLASSO on Silique_22 phenotype.

SNP	Genes	Annotation and functions
Chr1_7933107	AT1G22480	Expressed during mature plant embryo stage and expressed in plant embryo, seed
Chr1_16834866	AT1G44350	Expressed during plant embryo cotyledonary stage, expressed in carpel
Chr1_9777123	AT1G28050	Expressed during mature plant embryo stage
Chr3_2775964	AT3G09080	Expressed during mature plant embryo stage and expressed in plant embryo, seed
Chr3_15093392	AT3G43120	Expressed in plant embryo, carpel
Chr5_17242464	AT5G42980	Expressed in carpel
Chr5_10025947	AT5G28010	Expressed during plant embryo cotyledonary stage
Chr5_10025947	AT5G28020	Expressed in carpel, plant embryo
Chr5_18001211	AT5G44620	Expressed in plant embryo

SNP is the ID of the SNP from the dataset. The number after the dash is the position of the SNP. Genes denote the corresponding genes to the SNPs selected. Annotation and functions represent the functions of the associated genes.

**Table 15.** Genes and their annotations linked to the SNPs reported by BIGLASSO on Germ_22 phenotype.

SNP	Genes	Annotation and functions
Chr1_17913720	AT1G48460	expressed during mature plant embryo stage, expressed in seed
Chr1_17913720	AT1G48470	expressed during plant embryo cotyledonary stage
Chr4_10411003	AT4G19010	expressed during mature plant embryo stage,
Chr2_19474849	AT2G47450	expressed in plant embryo, seed
Chr5_17165413	AT5G42800	expressed during plant embryo cotyledonary stage
Chr4_17180542	AT4G36360	Expressed in plant embryo, seed
Chr4_17025614	AT4G35950	Expressed during plant embryo cotyledonary stage
Chr5_15766333	AT5G39400	Expressed in plant embryo
Chr3_2564078	AT3G08030	Expressed in plant embryo, seed
Chr1_6061258	AT1G17620	Expressed during mature plant embryo stage, plant embryo cotyledonary stage
Chr2_19439970	AT2G47380	Expressed in plant embryo, seed
Chr5_5912851	AT5G17880	Expressed in plant embryo, seed

SNP is the ID of the SNP from the dataset. The number after the dash is the position of the SNP. Genes denote the corresponding genes to the SNPs selected. Annotation and Functions represent the functions of the associated genes.

When AUTALASSO was applied to these categorical phenotypes, the resulting regression coefficients were all zero, indicating a failure to identify any SNPs. Further investigation revealed that AUTALASSO may have limitations with categorical phenotypes.

## 4 Discussion

In our experiments, we made several noteworthy observations regarding the SNP outputs of BIGLASSO and AUTALASSO. Notably, there was a distinct lack of consensus or overlapping SNPs between the two LASSO-based methods. This discrepancy may arise from the design of each method for different types of phenotypes. BIGLASSO demonstrates satisfying performance with most binary traits we have studied, and could effectively identify relevant SNPs, even when these are derived from originally categorical phenotypes. AUTALASSO is suited for quantitative traits but not binary or categorical based on our study. Therefore, we recommend to use it when the targets phenotypes are quantitative traits.

Another potential reason for the lack of consensus between the two LASSO-based methods can be the contributions of parameters in the two methods. Our choice to use default settings could have influenced the outcomes, suggesting that parameter tuning might yield different results. The exploration of this aspect demands further investigation.

Interestingly, when comparing BIGLASSO with PLINK, especially in the context of binary and categorical phenotypes, there was a remarkable agreement in their outputs. Such convergence can be leveraged as a validation metric for the reported SNPs. When multiple methods concur on specific SNPs, it amplifies our confidence in their potential causal relationship with the trait. This also opens the door for subsequent wet-lab validations to verify their contributions to the trait.

For the phenotype FT10, we observed a limited agreement between GWAS and BIGLASSO, but no agreement between GWAS programs and AUTALASSO. This discrepancy might hint at the complementary nature of LASSO techniques with GWAS in some cases. Some SNPs detected by LASSO-based methods might be overlooked by GWAS but could potentially influence the trait. Our validation of reported SNPs showed that some of them pointed to genes closely related to the phenotype. These findings suggest that combining LASSO-based methods with GWAS could provide a more comprehensive understanding of genetic associations. Since this study relies on experiments of one dataset, further research incorporating diverse datasets is needed to draw a comprehensive conclusion. Moreover, as discussed before, parameters play an important role in LASSO-based methods, they could also contribute to the disagreement of the results and are worth further study.

## 5 Conclusion and future work

In this study, we compared two LASSO-based methods with GWAS methods for finding significant SNPs. Experiments were conducted on an *A. thaliana* dataset with seven phenotypes, two binary, two quantitative, and three categorical.

This research highlights the potential of BIGLASSO and AUTALASSO to augment traditional GWAS by offering a more effective approach for uncovering elusive genetic markers within the context of the large-*p*-small-*n* dilemma. By showcasing the effectiveness of these computational techniques, our study bridges the gap between statistical models and machine learning in genetic analysis and sets a foundation for future research aimed at unraveling complex genetic relationships. The integration of these LASSO variants into GWAS represents a significant advancement in our toolkit for addressing the intricate challenges of genetic marker identification, contributing to the broader goal of enhancing precision and insight in the field of genomics.

There are several directions in which this study can be expanded to obtain better and more comprehensive results. Currently, only two LASSO-based methods are studied, but there are other similar methods that we could investigate, such as group-lasso and ridge regression. Additionally, parameters in these methods can be further studied to understand their influence on the experiments. Researchers can explore the possibility of creating a novel ensemble model by integrating LASSO and its variants. This ensemble approach has the potential to enhance the robustness and reliability of the results pertaining to significant genetic markers. Furthermore, researchers could focus on optimizing LASSO-derived methodologies for plant-specific characteristics (such as genomic variations and polyploidy) by developing specific pre-processing techniques like array customization of samples.

Currently, our study focused on the *A. thaliana*. Future studies could expand to other plant genomes or diverse phenotypes within each data type, expanding the scope and universality of our findings. Finally, we will investigate how to combine and rank SNPs output from different methods to enhance the accuracy and confidence of reported SNPs. This will enable the pipeline to synthesize and report significant SNP sets in a customized and reliable way. Researchers will have high confidence that these SNPs are truly associated with the phenotype and could even be good predictors for phenotype.

Researchers can also explore the integration of an independent and robust gold standard resource to validate the research findings effectively. Leveraging resources such as eQTL databases and established causal SNP datasets can serve as valuable benchmarks for validating the identified genetic markers and associations. This approach would further strengthen the reliability and applicability of the results obtained from LASSO-based methods and GWAS, providing a more comprehensive and validated understanding of genetic associations.

## Data Availability

The *Arabidopsis thaliana* dataset analysed in this study is available at https://easygwas.biochem.mpg.de/down/1/ [click the download button for AtPolyDB (call method 75, Horton *et al.*)]. The scripts and results for the SNP analysis can be accessed at https://github.com/DongdongHou006/LASSO-SNP.
